# Of Nature and Art in the Cure of Disease

**Published:** 1858-09

**Authors:** 


ART. II.—*Of Nature and Art in the Cure of Disease.* By SIR JOHN FORBES, M.D., D.C.L. (Oxon,) F.R.S.; Fellow of the Royal College of Physicians, Physician to the Queen's Household, etc., etc. Second edition. 12mo. pp. 261. New York: Samuel S. and William Wood, 1858.*

FOR the long period of fifty years, Sir John Forbes tells us, he has been actively engaged in the practice of medicine; and during much of that time he has been favorably and extensively known by his many able contributions to medical literature. As editor of the *British and Foreign Medical Review*, his essays, reviews, and criticisms were characterized by candor and ability, and not until his celebrated article on "Homœopathy, Allopathy, and Young Physic," in 1845, were the soundness of his views called in question by any considerable portion of the profession. That essay gave rise, at the time, to much controversy, and was considered by many as anything but orthodox. It must be conceded, however, that it reflected the opinions of many practical men in both hemispheres. Every one knows that a new life has been gradually infusing itself into the ranks of the profession. Within the last twenty years a revolution has passed over the face of Old Medicine; and this, if not caused by, has taken place at least coincidently with, the unparalleled advances in the knowledge of the pathology and diagnosis of diseases. To-day clinical medicine is far in advance of our elementary treaties on the practice of medicine, and

---

* This is the London edition with an American title page, and to Messrs. Wood is great credit due for the very good habit they are falling into of thus presenting us with foreign works.

most sincerely do we thank Sir John Forbes for much of the reformation which has, within the last twelve years, been wrought in the *drugging system.* He has brought to bear, his experience, his learning, and his reputation, against a mischievous polypharmacy, and has endeavored to strengthen the confidence, at least, of the junior practitioner in the powers of nature, to arrest the processes of disease. In this spirit, he desired the present work to be regarded in the light of "*A Legacy to his Younger Brethren;*" and if it only lead to the zealous cultivation of the subject of which it treats, the result, he thinks, cannot fail to be of inestimable value to the cultivators.

"For," he adds, "on the profounder, more critical, and purer study of Nature, as manifested in disease, rest, in my judgment, the best hopes of improvement in the medical art; and to this study the spirit of my book may, at least, lead the way and give the initiative, if its actual contents are found of lesser importance."

Want of trust in the powers of Nature to cure disease, and too much faith in remedial agents as the sole means of cure, are, in the estimation of the author, the great hindrances to the progress of a safe and rational practice. This error, which has so deeply tainted our medical literature, is combatted throughout the entire work, and the conviction is expressed that it has its origin in ignorance of natural laws governing disease. Hence the first chapter, the starting point of Sir John Forbes's theory of therapeutics, treats of the NATURAL HISTORY OF DISEASES; which very justly forms, in his estimation, the only sure foundation of a scientific knowledge of the medical art.

"Such has ever been the want of trust in Nature, and the over-trust in art prevalent among the members of the medical profession, that the field of natural observation has been, to a great extent, hidden from them; hidden either actually from their eyes, or virtually from their apprehension. The constant interference of art, in the form of medical treatment, with the normal processes of disease, has not only had the frequent effect of distorting them in reality, but even when it failed to do so, has created the belief that *it did so;* leading, in either case, to an inference equally wrong; the false picture, in the one instance, being supposed to be true; the true picture, in the other, being supposed to be false."

The first half of the volume is devoted mainly to setting forth his views on the nature, origin, causes, mode of termination, course, and treatment of diseases, and especially "how they comport themselves when left undisturbed by any extrinsic artificial influence." The second half of the work attempts to do for art what the first does for nature, viz.: "to exhibit the true character of the medical art, its exact relations to the natural conditions of diseases, and the extent of its power to modify and cure them." In the first division he labors to establish the fact that Nature's power to cure disease "exists in a degree *beyond* the ordinary belief of the public and the younger members of the profession;" and in the second, to establish the correlative fact of the power of art to cure disease, "being greatly *below* the same standard of belief."

The author's general notions of disease are clearly and concisely expressed, and, in an *à priori* sense, furnish strong grounds for many of his therapeutic deductions. He regards the morbid condition constituting disease, whether dynamic, structural, or humoral, as the immediate result of the vital actions *ordinarily existing in the system;* he affirms that there is no *new* thing superadded to the living body, and constituting a special entity; that causes of disease, in short, as ordinarily understood, are not real or efficient causes at all; they merely constitute "the *occasions* on the *prompting* of which the natural or vital functions of the living body set about forming the diseases themselves." It will be thus seen that he regards

"All morbid action as but a modification or perversion of some natural or normal action or function; and all the physical results constituting morbid structural alterations, are mere perversions or modifications of natural or normal textures; or, at most, analogous textures fabricated from the same materials by like processes."

Having proved, at least to his own satisfaction, that diseases are *natural,* though not normal conditions of the human body, and that they are formed and maintained by the same vital powers which regulate and constitute the ordinary conditions of health, he urges, as a legitimate deduction from his premises, the reasonableness and probability of the natural cure of diseases; for, he adds,

"If nature, without any extraneous aid, either dynamical or material, can build up diseases, there would seem no substantial reason why she should not be equally able to effect their removal."

The author, however, we think, exhibits a want of candor, or seeks to give himself undue prominence, in his frequent allusion to the study of the *Natural History of Diseases.* Its importance he surely cannot over-estimate. It is the pedestal upon which the whole superstructure of a sound medical education must rest. But Sir John appears to lose sight of, or to ignore, the fact that the whole tendency of modern medical science is in that direction. There never was a time in the history of medicine when more attention was given to the associated study of Physiology and Pathology. The study of these branches is urged by our National Medical Association; they are taught in every respectable medical college in our land. Everywhere, both in the Old World and New, zealous workers are in this field, searching, by scalpel, by microscope, by weight, and test-tube, and analyses, into the mode by which the normal changes of the body are perverted in the infinite deviations or alterations which constitute disease; thereby attempting to arrive at just such a history as the author shadows forth.

But the autocracy of Nature, as the author expresses it, in the *cure* of these diseases, is quite another question. Upon this point, as yet, an honest difference of opinion may exist. It must be conceded, however, that the cures of all diseases are affected by nature; art is only her assistant, and cures by her means. In visible diseases, surgical so called, no body doubts that this is the case. Every surgeon admits that it is not he who cures a fracture, a wound, or an ulcer, but it is Nature; the vital power, which restores through her wonderful operations of expelling foreign matter, throwing out fresh bony material, binding up by means of her natural splints, and uniting soft parts through the mysterious organization of the "neucleated blastema" of the blood. The office of the surgeon here is merely to guide these operations properly and to remove obstacles. And the same principle holds good in internal diseases, the relations of which are hidden from our senses.

The practical question, then, which presents itself, is, What can Nature do unaided? Can we rely exclusively upon the natural powers to re-establish the lost balance of the system? Can Nature do it in the most effective and expeditious way if left to herself? Or can she be assisted by Art? We have already conceded the power of Nature, and we express our honest conviction when we further add, that in many diseases Nature will effect a cure better and more expeditiously if left to herself; in some she is manifestly aided by Art—Art, it may be, rendering the internal cure *possible;* in others Nature succeeds at once to the powerful influences of a morbid poison, which, if neutralized by a proper antidote, would enable the natural life-forces of the system again to assert their control. Thus, Art can sometimes take away the whole disease by removing the exciting cause, and thus dispense with the curative reaction of Nature for its removal. Then, again, Nature may not have sufficient power to perform the internal curative process. Here Art interferes by suitable tonics, stimulants, and strengthening remedies. Art can also remove obstacles which render the curative process of Nature difficult or impossible, or may support Nature by combatting particular forms of disease by appropriate remedies. Who can doubt, for instance, the power of quinine in arresting the paroxysms of the malignant intermittents of the Mississippi Valley, and thereby, in hundreds and thousands of instances, furnishing, so far as we know, the only hope of recovery? Or, who can question the beneficial effects of the salts of iron on the blood in chlorotic anæmia? We might multiply many such instances. Our opulent materia medica is full of them.

But, although a powerful advocate of the regiminal treatment of disease, Sir John Forbes by no means despises or rejects the use of drugs. Thus, in speaking of alteratives, he says, "the class contains some remedies of positive and evident power;" he lauds opium as "the noblest instrument of the medical art." Indeed, under the head of the "Instruments of the Medical Art," he scarcely passes in review a single class of remedies of which he does not speak more or less favorably; and we are at a loss to know how an honest, conscientious physician, who believes he can command a remedy of "obvious and admirable power," can refuse his patient its benefits for the purpose of testing whether the unaided powers of Nature are sufficient to remove disease. We once had an opportunity of studying "the natural history of disease" in a case of paroxysmal fever of the West. It was, in the onset, a simple uncomplicated case of *malarious fever*. We had seen such diseases treated by pepper and steam, by cold baths and warm, by emetics, cathartics, quinine, arsenic, strychnine, and "Indian chologogues;" but never before by infinitesimal doses of *sugar!* We made accurate note of it at the time, and published it for the benefit of those interested in the study of the *natural history of disease.* The patient—a valuable citizen—died. On his tombstone might have been appropriately written, "*Died for the want of thirty grains of quinine—a victim to the dreamy speculations of Homœopathy.*" We had, in this case, it is true, the rare opportunity of studying "the natural history of the disease," but unhappily our knowledge was imbittered with the thought that it was at the expense of valuable life.

The enlightenment of the profession has established too clearly the fallacy of relying exclusively on the morbid evolutions of Nature for the cure of disease. We find her practices too often baleful, and often she deals with us in a double sense. While she "soothes the grief of a wound" by pouring out serum, and knits it up by her adhesive inflammation, by the very same method she contracts the valves of the heart, effectually closes the glottis, or glues the intestines into fatal entanglements. She pours out the serum of the blood on her free surfaces, and we bless her for her recuperative powers in saving us from dropsy; while another poor victim dies because she deluged his heart with the same fluid. One glorifies her for saving him from apoplexy by an epistaxis or hemorrhoidal flux; while another dies because she poured the same fluid into his lateral ventricles. These errors of pathological Nature lead us to distrust a theory or system based exclusively on her natural reactions.

But it would be doing the author injustice to assert that such is his creed. He evidently has no leanings toward homœopathy. While he values the negative statistics, as evidence of Nature's unassisted powers, he pronounces the system itself "one of the greatest and most singular delusions that has ever been entertained by the professors of the healing art." And, in comparison with a true and rational practice, adds,—

"Its results are greatly superior to those of Homœopathy, which, in its blind devotions to the most visionary hypothesis, ignores and overlooks the most positive and obvious means of aid presented by the resources of ordinary medicine."

The reader may desire to know how Sir John Forbes reconciles the apparent contradiction involved in his almost unlimited faith in the powers of Nature to cure disease on the one hand, and his laudation of the more valuable articles of our materia medica on the other. We quote his own words, premising that, in discussing the various modes of treatment that have been and are in vogue, he advocates what he calls RATIONAL EXPECTANCY.

"This modification of the indirect physiological method of treating diseases (more especially acute diseases) I regard as at once the most philosophical, the safest, the surest, and the most successful of all the forms it assumes in practice. Although in appearance it differs little from the last form of treatment, except in degree, it is based on a somewhat different principle, and seeks to fulfil different indications. In the first place, it completely recognizes the autocracy of nature in the cure of acute diseases; and proceeds on the principle that it is not only useless, but injurious, to attempt to suppress, or greatly to modify the morbid processes by strong measures of a perturbative or exhaustive kind.

"The indications which this mode of treatment seeks to fulfil are chiefly the following:—1st. To place the diseased body in the most favorable circumstances for the development and exercise of its own conservative powers, by the institution of a proper regimen, in the most comprehensive sense of that term. 2d. To endeavor thereby, or through the use of medicaments, to remove such obstacles to the favorable action of the conservative and restorative powers, as may be removable without the risk of checking or injuriously perverting them. 3d. Applying these measures under a watchful supervision; not to attempt, by any vigorous measures, to alter the course of the morbid processes so long as they seem to keep within the limit of safety; and when they transgress this limit, only then to endeavor to modify them by such mild measures as, if they fail of doing good, cannot do much harm. 4th. To be on the watch against possible contingencies, which may demand the employment of measures of exceptional activity, whether in the form of regimen or medicine, and, when required, to apply such measures with the necessary vigor."

We most heartily concur in Sir John Forbes's method, so far as he combats the purely empirical, as contra-distinguished from the physiological mode of treating disease, and we doubt not he is doing a good work in waging war against the reckless in the indiscriminate use of drugs. In a practical point of view, two errors are apt to exist, or rather two extremes, both of which the intelligent and conscientious physician should carefully avoid. The first is doing too little—the negative treatment which leaves all to nature; the second is that of doing too much. In the effort to *cut short,* as it is called, or extinguish disease by a "bold stroke," the organism too frequently suffers more from the administration of the remedies than from the disease. This indication is, however, happily less acted on now than formerly; and if Sir John has had much intercourse with physicians of less than twenty years standing, and is familiar with their practice, he must have observed the growing tendency to scepticism in the heroic dosing of the olden time. It is an unfortunate constitution of the human mind that it inclines to go from one extreme to another in an opposite direction. In avoiding the Scylla of ancient dogmatism, we may plunge into the Charybdis of a false modern scepticism—a scepticism which, having no positive basis for its opinions, is blown about by every wind of public opinion. Such men are the clogs to the profession, and become, sooner or later, victims of their own incredulity. "I know of no more revolting position," says Renouard, "for a conscientious man, nor a more ridiculous one, than that of a physician who has no confidence in the means he employs. Such a man cannot possibly pursue the study and the practice of his art with the zeal, application, and assiduity which alone can secure him real honest success."

But there is another and a philosophical scepticism of a widely different kind—a scepticism which doubts but to confirm and prove, which waits upon, and follows the lead only of well-established fact, and which co-exists with an ardent love for the profession, and hope for its future. Such a scepticism has ever characterized the most enthusiastic and successful cultivators of science; and, with all his doubts, it will be seen by the following extract, that Sir John Forbes has no mean opinion of our art:—

"According to the lowest estimate that can be justly formed of the medical art, it must still hold its pre-eminence as one of the greatest boons that human intellect has ever elaborated for the benefit of man's estate. With all its feebleness and all its uncertainties, it possesses, and ever must possess, a sufficiency of solid truth and solid power to make it worthy of the study and pursuit of the noblest intellects and tenderest hearts."

We have thus attempted to give, if not a detailed summary, at least the spirit of Sir John Forbes's book, and most heartily and sincerely do we commend it to the profession of America. It is a valuable "legacy" to all who are honestly seeking for truth in their profession; and, although extreme in some of his views, he has evidently written in an earnest hatred of quackery in all its forms, and in an honest desire to promote the highest interest of our common profession. And such, we think, must be its tendency. We have read and re-read it, we hope, with profit; and while our faith in the powers of Nature to cure diseases has been strengthened, we no less continue to believe that "*the Lord hath created medicines out of the earth, and he that is* WISE *will not abhor them.*"

