# Correction

**Published:** 1858-11

**Authors:** 


CORRECTION.—In our last issue, in noticing Dr. Forbes's "Nature and Art in the Cure of Disease," it is mentioned in the foot-note that "this is the London edition with an American title-page." We must, however, correct our mistake, and give due credit to Messrs. S. S. & W. Wood, in saying that the work is wholly American in its getting up.

